# Removal of astringency from persimmon paste via polysaccharide treatment

**DOI:** 10.1016/j.heliyon.2022.e10716

**Published:** 2022-09-21

**Authors:** Yoko Tsurunaga, Tetsuya Takahashi, Mina Kanou, Misaki Onda, Mika Ishigaki

**Affiliations:** a Faculty of Human Science, Shimane University, 1060 Nishikawatsu-cho, Matsue City, Shimane 690-8504, Japan; b Graduate School of Human and Social Sciences, Shimane University, 1060 Nishikawatsu-cho, Matsue City, Shimane 690-8504, Japan; c Faculty of Education, Shimane University, 1060 Nishikawatsu-cho, Matsue City, Shimane 690-8504, Japan; d Institute of Agricultural and Life Sciences, Academic Assembly, Shimane University, 1060 Nishikawatsu, Matsue City, Shimane 690-8504, Japan

**Keywords:** Persimmon paste, Polysaccharide, Astringency, Tannin, Soluble tannin content, Syneresis

## Abstract

Non-astringent persimmon (*Diospyros kaki* Thunb.) paste is typically produced by treating astringent persimmon fruit with alcohol or dry ice (to remove tannins) followed by abrasion. However, considering the large yield of astringent persimmons harvested in a short time, this long, laborious method has hindered the use of persimmon paste in food processing. Herein, the addition of polysaccharides was used to produce a non-astringent persimmon paste while maintaining its quality. Among the nine evaluated polysaccharides, high- (HM) and low-methoxyl (LM) pectins, carrageenan, xanthan gum, and sodium alginate exhibited high astringency removal efficiencies. No astringency recurrence was observed after freezing when HM or LM pectin, guar gum, carrageenan, or sodium alginate were added. Moreover, the addition of HM pectin, or LM pectin, or sodium alginate prevented astringency upon heating. Additionally, guar, xanthan, tara gum, or carrageenan effectively inhibited syneresis. Thus, high-quality pastes could be easily and efficiently produced using a combination of polysaccharides.

## Highlights

• Easy and efficient method to remove astringency from persimmon paste was devised.
• HM and LM pectin, carrageenan, xanthan gum, and sodium alginate removed astringency.
• Guar gum, xanthan gum, tar gum, and carrageenan effectively inhibited syneresis.
• Addition of HM or LM pectin or sodium alginate prevented astringency upon heating.
• Astringent persimmons can be rapidly treated during a short harvest period.

## 1 Introduction

Persimmons (*Diospyros* sp*.*) are marketed in astringent (containing soluble tannins) and sweet (containing insoluble tannins) varieties. Although astringency may be removed via treatment with carbon dioxide gas, dry ice, or alcohol (Cortes et al., 2017; Yamada et al., 2002; Salvador, et al., 2008), these treatments are long and laborious, and thus challenging to implement in the food industry, as rapid processing of large quantities of astringent persimmons during the short harvest period is required. Fruit paste is a crucial processed product used in the production of ice cream, cakes, cookies, etc. However, the production of non-astringent persimmon pastes by grinding persimmons subjected to an astringency removal treatment is laborious. While astringency removal via dry ice or alcohol treatment is effective, astringency recurrence is sometimes observed after heating, hindering the development of certain processed products. Herein, we hypothesized that polysaccharides could be used to remove tannins from astringent persimmon paste (AP) and evaluated the practical utility of this method.

Soluble tannins may form complexes with polymers such as proteins and polysaccharides, e.g., the interactions of soluble tannins with human salivary proline-rich proteins (Canon et al., 2013; Poncet-Legrand et al., 2006; Freitas and Mateus, 2002; Lu and Bennick, 1998; Mehansho et al., 1987; Murray et al., 1994; Soares et al., 2018) and the use of soluble tannins to remove proteins from sake and wine (Matsuo and Ito, 1978; Pizzi, 2019). Phenols are the key tannin components, and their interactions with proteins depend on temperature, pH, protein type, concentration, phenol (i.e., tannin) type, and structure (Ozdal et al., 2013). Pectin, a polysaccharide, forms complexes with tannins (Watrelot, et al., 2013) and galloyl-type catechins (Hayashi et al., 2005). Complex formation between tannins and polysaccharides is mainly controlled by cooperative hydrogen bonding and hydrophobic interactions (Mamet et al., 2018), and SEM analysis revealed that their association is due to intermolecular crosslink formation between the carboxyl groups of pectin molecules and phenolic hydroxyl groups of tannins (Mamet et al., 2017). However, the use of polysaccharides to remove the astringency of persimmon paste remains unexplored. Therefore, we herein (i) evaluate nine polysaccharides typically used as gelling agents or thickeners in the removal of the astringency of persimmon pastes via the formation of polysaccharide-tannin complexes and (ii) examine the effects of heating or freezing on the astringency of polysaccharide-treated tannin paste (PAPP). Soluble tannin content (STC) is used as an astringency indicator, whereas color and syneresis are used as paste quality indicators. The mechanism of formation of polysaccharide-tannin complexes was further investigated using Fourier transform infrared spectroscopy (FT-IR) and Fourier transform near-infrared spectroscopy (FT-NIR).

## 2 Materials and methods

### 2.1 Materials

The adjustment of persimmon paste was done using a previous report (Tsurunaga et al., 2021) as a reference. Astringent Saijo persimmons harvested at the Shimane Prefectural Agricultural Technology Center (Shimane, Japan) in November 2017 were ground to a smooth paste using an Osterizer 16-speed blender with a capacity of 1 L (Oster, Boca Raton, FL, USA). Low-methoxyl (LM) pectin (JP-20), carrageenan (V-120), guar (GR-10), xanthan (V-10), gellan (GP-15), and tara (type A) gum were provided by Ina Food Industry (Nagano, Japan). High-methoxyl (HM) pectin (from *Citrus* sp*.*) was purchased from Nacalai Tesque (Kyoto, Japan), and sodium alginate (first grade) was purchased from Wako Pure Chemical (Osaka, Japan). Gelatin (G-3153E, Nitta Gelatin, Osaka, Japan) was used as a protein for comparison.

### 2.2 Sample preparation

When the effect of the added polysaccharides (1, 3, 5, or 10 % [w/w]) on the AP was being investigated, astringency removal was observed for several polysaccharides, even at the lowest percentage of 1 % (w/w). Therefore, a polysaccharide percentage of 1 % (w/w) with respect to the total weight was used in subsequent studies, with mixtures homogenized using the blender.

### 2.3 Thermal treatment

Heat treatment was carried out using the previous report (Tsurunaga et al., 2021) as a reference. To mimic the treatment commonly used to sterilize pastes in the food industry under normal pressure, we placed pristine AP (50 g) in a heat-resistant bag (B-1318, Meiwa Pax, Osaka, Japan) and heated it at 100 °C for 40 min using an induction heater (MR-B20, Toshiba, Tokyo, Japan). In addition, a 50 g AP sample was enclosed in an aluminum pouch (AL14, Seisan Nipponsha, Fukuoka, Japan) and heated at 121 °C in an autoclave (BS-245, Tomy Seiko, Tokyo, Japan) for 4 min to mimic the standard conditions used to sterilize foods in retort pouches.

### 2.4 Freezing

For freezing, which was examined because fruit pastes are typically distributed in frozen forms, each paste sample was placed in a plastic bag (B-1318, Meiwa Pax) and stored at −25 °C for three months.

### 2.5 External appearance and syneresis

To determine the effects of polysaccharide addition and heating/freezing on AP appearance, each paste sample was placed in a stainless-steel dish (40 × 15 mm [diameter × height]) and photographed using a digital camera (WG-40W, Ricoh, Tokyo, Japan). To observe syneresis (i.e., the loss of liquid from the gel matrix), a 15 g paste sample (mass = *W*_0_) was placed in a 50 mL tube, subjected to 5 min of centrifugation (50B-7, Sakuma Manufacturing, Tokyo, Japan) at 3000 rpm, and photographed using the camera. The supernatant was carefully collected using a Pasteur pipette, and its mass (*W*_*1*_) was measured to determine the extent of syneresis calculated as 100 % × *W*_*1*_/*W*_*0*_ (Sanchez et al., 2010).

### 2.6 Physicochemical evaluation of astringency

STC was used as an astringency index because of its high correlation with organoleptic (sensory) astringency in humans. For STC determination, a 5 g paste sample was dispersed in 80 % (v/v) methanol and appropriately diluted. STC was measured using the Folin-Ciocalteu method, and the obtained data were analyzed using a modification of the method proposed by Chung et al. (2015). The water content of the pastes from each treatment was measured, and it ranged from 78.2 to 78.9 %, that is, it was almost identical. In addition, reports examining the astringency of persimmon fruit and paste are shown per fresh weigh (Amorim et al., 2020; Cortes et al., 2017). Since the condition of the paste during distribution is also in the form of a paste (fresh), it was decided to present the results of this study per fresh weight of STC, and STC was expressed as milligrams of (+)-catechin equivalent/100 g of fresh fruit (mg CTC eq/100 g FW).

### 2.7 Fourier transform infrared spectroscopy (FT-IR)

The FT-IR spectra were recorded in an A-Cary 630 FT-IR spectrometer (Agilent Technologies, Los Angeles, USA) with 4000 to 400 cm^−1^ as the wavenumber and 2 cm^−1^ as the resolution accuracy (Higgins and Rein, 2011). A horizontal attenuated total reflectance device (ATR) was used for measuring the spectra of the persimmon paste.

### 2.8 Fourier transform near infra-red spectroscopy (FT-NIR)

An IRTracer-100 system (Shimadzu, Kyoto, Japan) was used for NIR measurement. The optical path length of an open synthetic quartz cell was 0.05 mm. The wavenumber region measured was in the range of 10000–4000 cm^−1^, the spectral resolution was 4 cm^−1^, and 256 spectra were collected. The temperature of the quartz cell was controlled at 25 °C by a Mid-IR base (PN 111-40XX, Pike, Cottonwood, USA) with a digital temperature controller (PN 076–1510, Pike). The stability of the temperature was ±0.5 °C.

### 2.9 Water content

The moisture content was measured using a digital analyzer MS-70 (A&D Co., Ltd., Tokyo, Japan) (Wada et al., 2016). Samples were spread thinly on glass fiber sheets and measured at a set temperature of 160 °C.

### 2.10 Statistical analysis

Data were statistically analyzed using SPSS software (Version 25.0, SPSS, Chicago, IL, USA), and the results were expressed as means ± standard errors. Moreover, for multiple comparisons, data were evaluated using one-way analysis of variance (ANOVA) followed by Tukey’s test.

## 3 Results and discussion

### 3.1 Effect of polysaccharide addition on the STC of AP

**Figure 1:**
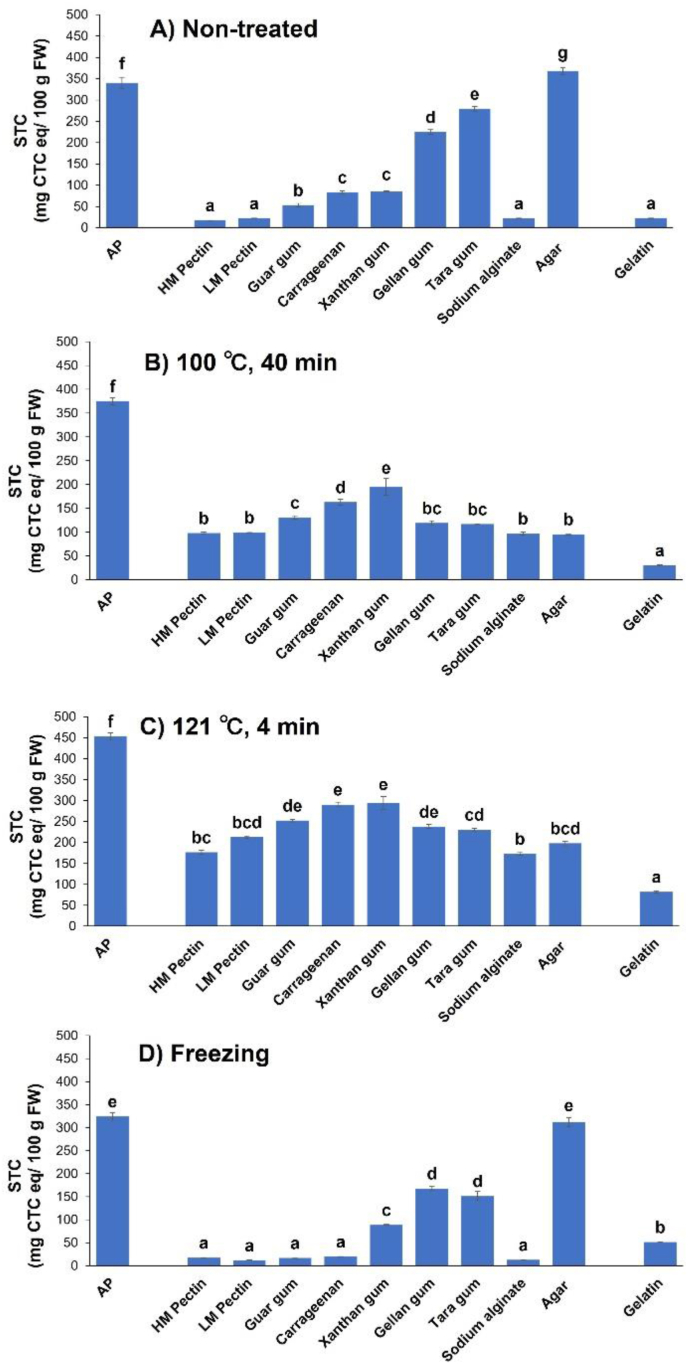
Effects of polysaccharide addition on the soluble tannin content (STC) of astringent persimmon paste (AP) subjected to (A) no treatment, heating at (B) 100 °C or (C) 121 °C, or (D) freezing. Vertical bars represent mean values with standard errors (*n* = 6). Letters indicate significant differences (*P* < 0.05). HM, high-methoxyl; LM, low-methoxyl.

Figure 1A shows the effects of polysaccharide addition to the STCs of non-treated samples. AP with no additives was used as the negative control, whereas gelatin, which reduces astringency, was used as the positive control (Obreque-Slier et al., 2010). The STC of pristine AP was 339.5 ± 9.8 mg CTC eq/100 g FW, which decreased to 22.9 ± 1.6 mg CTC eq/100 g FW after gelatin was added, which demonstrates the good astringency removal property of gelatin. The STCs of PAPP ranged from 17.4 ± 0.4 to 367.7 ± 7.8 mg CTC eq/100 g FW, with the lowest STCs (i.e., highest astringency removal efficiencies) observed for HM (17.4 ± 0.4 mg CTC eq/100 g FW) and LM (22.1 ± 0.3 mg CTC eq/100 g FW) pectins, carrageenan (83.6 ± 7.6 mg CTC eq/100 g FW), guar (52.7 ± 10.7 mg CTC eq/100 g FW) and xanthan (84.7 ± 3.1 mg CTC eq/100 g FW) gum, and sodium alginate (21.7 ± 0.9 mg CTC eq/100 g FW). These results are equivalent or superior to those obtained for gelatin. Given that the paste did not exhibit an astringent taste at an STC of <100 mg CTC eq/100 g FW (Yamada et al., 2002; Amorim et al., 2020; Del Bubba et al., 2009; Inari and Tomoyeda, 1992; Noypitak et al., 2015; Taira, 1996), HM and LM pectins, guar and xanthan gum, carrageenan, and sodium alginate may remove astringency from AP well.

The astringent taste of AP may be caused by proanthocyanidins, i.e., condensed tannins (Tanaka et al., 2010; de Freitas et al., 2003). Although the complexation of proteins by condensed tannins was previously investigated (Dinnella et al., 2009; de Freitas et al., 2003), little is known regarding the complexation of condensed tannins by polysaccharides. For example, the binding of pectin by condensed tannins involves hydrophobic interactions and hydrogen bonding (Watrelot et al., 2013, 2014). Pectin, xanthan, and guar gum also inhibit protein/tannin precipitation (Wang et al., 2020), and an investigation of Arabic gum, pectin, and polygalacturonic acid showed that pectin was the most effective inhibitor of this precipitation (Soares et al., 2012). Thus, several polysaccharides may bind to condensed tannins to reduce astringency.

### 3.2 Effect of heating on the STC of PAPP

For all samples, the STCs after heating at 121 °C for 4 min (Figure 1C) exceed those obtained after heating at 100 °C for 40 min (Figure 1B). Therefore, processing temperature affects the STCs more strongly than processing time does. After heating at 100 °C for 40 min, the STCs of the pristine and gelatin-containing APs were 374.7 ± 7.5 and 29.7 ± 1.3 mg CTC eq/100 g FW, respectively, i.e., gelatin addition prevents the reappearance of astringent flavor upon heating. The STCs obtained after the addition of each polysaccharide were lower than that of AP, i.e., similarly to gelatin, the polysaccharides prevent astringency recurrence after heating. STCs of 97.9 ± 2.4, 99.1 ± 1.6, 96.9 ± 3.6, and 95.2 ± 1.4 mg CTC eq/100 g FW were observed for the samples containing HM or LM pectin, sodium alginate, or agar, respectively, after heating at 100 °C for 40 min. This indicates that as an astringency recurrence inhibitor, gelatin is superior to polysaccharides. Upon heating at 121 °C for 4 min, STCs of 453.5 ± 22.0 and 82.2 ± 2.5 mg CTC eq/100 g FW were observed for the pristine and gelatin-containing APs, respectively, i.e., gelatin effectively inhibits astringency recurrence, even at 121 °C. In the case of PAPP, HM pectin and sodium alginate were the most effective inhibitors; however, their respective STCs of 176.5 ± 3.9 and 173.1 ± 4.6 mg CTC eq/100 g FW exceed that obtained for gelatin under the same conditions and are also higher than the STCs obtained using the same polysaccharides after heating at 100 °C for 40 min. Therefore, although the polysaccharides used inhibit astringency recurrence upon heating, none outperform gelatin.

### 3.3 Effect of freezing on the STC of PAPP

Figure 1D shows the effect of freezing on the STC of PAPP. After freezing, the pristine and gelatin-containing APs exhibit STCs of 324.0 ± 7.9 and 51.1 ± 0.8 mg CTC eq/100 g FW, respectively. The latter value exceeds that of the non-frozen gelatin-containing sample (22.9 ± 1.6 mg CTC eq/100 g FW, Figure 1A), i.e., the STC increases after freezing, which is ascribed to the concomitantly weakened binding of soluble tannins due to gelatin denaturation. The STCs of the frozen PAPP samples containing HM or LM pectin, guar gum, carrageenan, or sodium alginate were 17.1 ± 0.7, 11.6 ± 0.5, 16.6 ± 0.9, 19.7 ± 0.4, and 12.6 ± 0.5 mg CTC eq/100 g FW, respectively, which are similar to the STCs of the respective non-frozen samples (Figure 1A), indicating that astringency recurrence after freezing was inhibited. For xanthan gum, the STC increased from 84.8 ± 1.3 (Figure 1A) to 88.7 ± 1.1 (Figure 1D), and for guar gum and carrageenan, the STCs decreased from 52.7 ± 4.4 and 83.6 ± 3.1 mg CTC eq/100 g FW (Figure 1A) to 16.6 ± 0.9 and 19.7 ± 0.4 mg CTC eq/100 g FW (Figure 1D), respectively, after freezing. The decrease in STC after freezing may reflect the concomitant formation of new complexes. We could not clarify the underlying mechanism in this study, and this is a subject for further investigation. Based on these results, astringency inhibition should be retained after freezing.

### 3.4 Spectroscopic analysis of astringency reduction by FT-IR and FT-NIR

In this study, spectroscopic analysis was carried out using FT-IR and FT-NIR to investigate the mechanism of the astringency reduction using polysaccharides at a molecular level. As a polysaccharide added to AP, guar gum was selected because of its high effects on astringency reduction and syneresis suppression.

**Figure 2:**
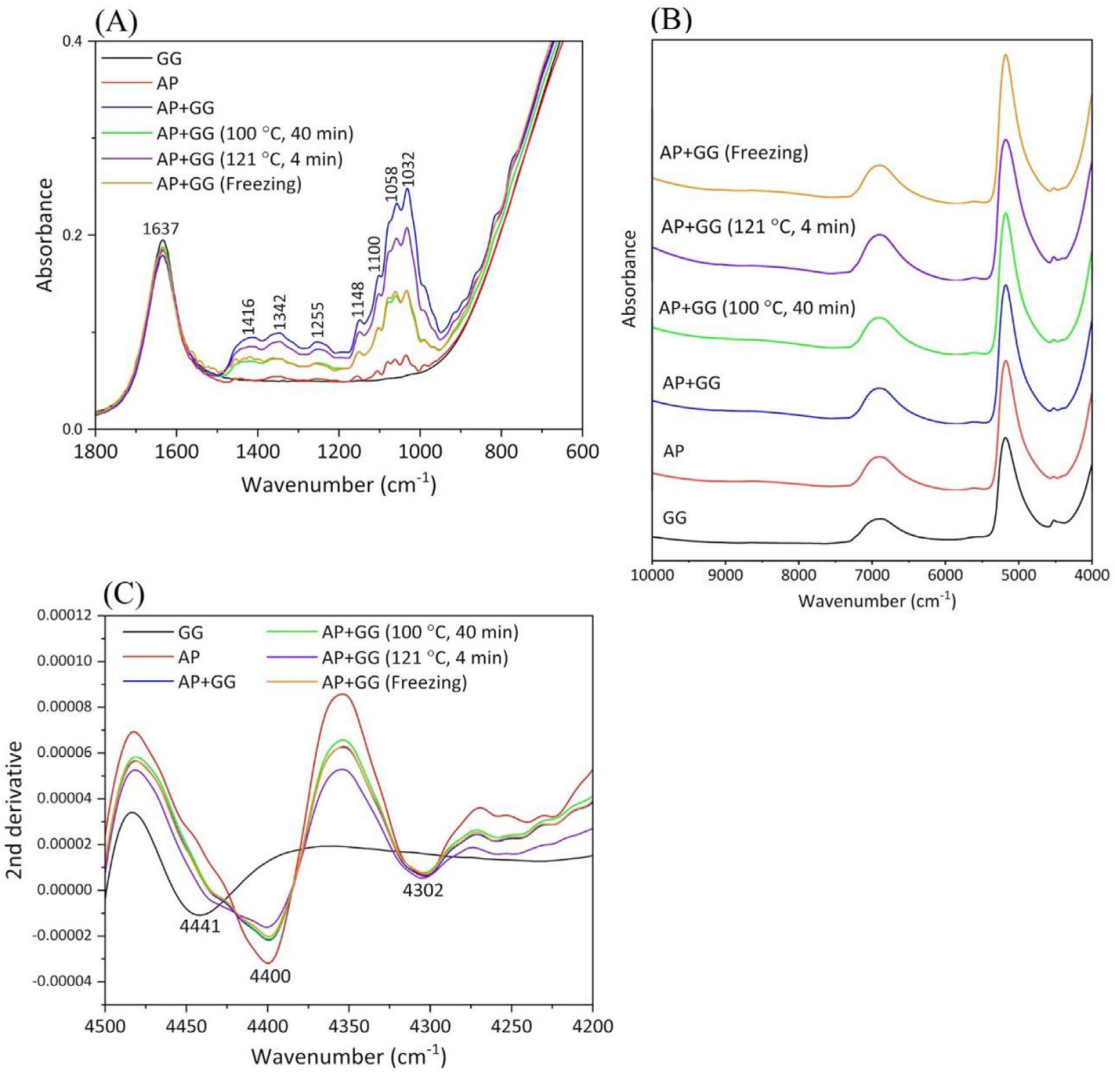
(A) IR absorbance spectra in the 1800–600 cm^−1^ region (B) NIR absorbance spectra in the 10000–4000 cm^−1^ region and (C) their second derivative spectra in the 4500–4200 cm^−1^ region. AP, astringent persimmon paste; GG, guar gum.

Figure 2A shows IR absorbance spectra in the 1800–600 cm^−1^ region obtained from guar gum aqueous solution, AP, and AP with guar gum. The broad band at 1637 cm^−1^ is mainly due to the O–H bending mode of water. The bands at 1148, 1100, 1058, and 1032 cm^−1^ are assigned to the C–C and C–O stretching vibrations; the band at 1255 cm^−1^ is derived from the C–C_6_H_5_ stretching vibration; and, the bands from C–H deformation were detected at 1416 and 1342 cm^−1^ (Movasaghi et al., 2007; Pantoja-Castro and González-Rodríguez, 2011). These spectral features of AP, strongly reflecting the contributions from C–C, C–O, and C–H components, are reasonable because a tannin monomer has aromatic rings with many phenolic hydroxy groups. When guar gum was added to AP (AP + GG), these band intensities tended to increase. This result is likely to be derived from a complex formation occurring between tannins and guar gum. When tannins form complexes with polysaccharides, tannins are expected to form condensed tannins with new C–C and ether bonds. Thus, IR spectra were likely to reflect the molecular structural changes of tannins following the addition of guar gum. Furthermore, by heating, freezing, or adding guar gum, these band intensities derived from C–C and C–O components decreased compared to those without the heat treatments.

This is because these new bonds in the complexes were partially dissociated due to the influence of heating or freezing.

Figure 2B shows the NIR absorbance spectra in the 10000–4000 cm^−1^ region obtained from the same sample group. Though the broad features due to water were observed at 6900 and 5200 cm^−1^, characteristic bands derived from persimmon, except for their moisture, were indistinguishable. Thus, the second derivative spectra were calculated to separate small bands based on the variations of the spectral curvature. The absorbance bands were identified as negative peaks in the second derivative spectra. To investigate the spectral differences caused by the addition of guar gum, as suggested by IR analysis, the second derivative spectra in the 4500–4200 cm^−1^ region were focused (Figure 2C). Two bands at 4400 and 4302 cm^−1^ were mainly detected in the spectra obtained from samples including AP, and they were assigned to the combination of O–H and C–O stretching modes and C–H stretching and bending modes, respectively (Weyer, 2007). Although the band intensity at 4302 cm^−1^ remained unchanged upon the addition of guar gum, the one at 4400 cm^−1^ decreased with the addition of guar gum. This result indicates that phenolic hydroxyl groups of tannind are linked with polysaccharides and that the band intensities from O–H groups are expected to decrease. That is, IR and NIR results successfully revealed the mechanism of astringency reduction to be caused by tannins condensation and the formation of complexes with polysaccharides.

### 3.5 Effect of polysaccharide addition on AP syneresis

**Figure 3:**
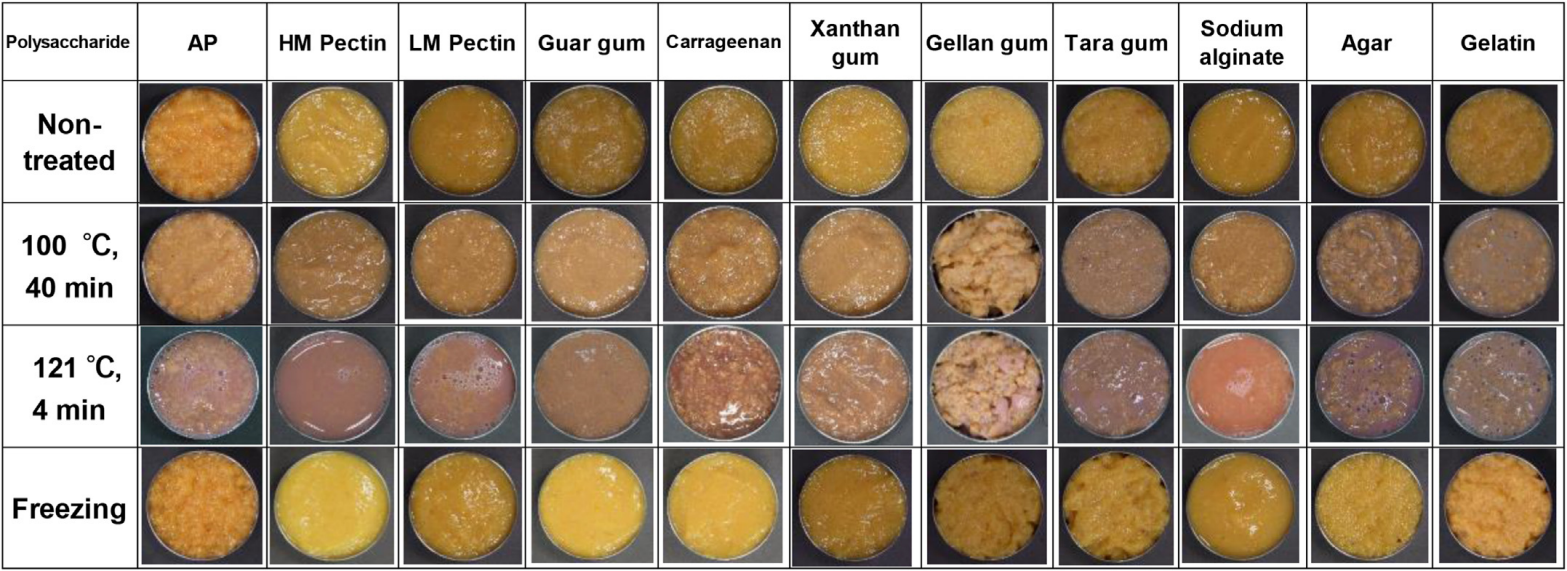
Effects of polysaccharide addition and heating/freezing on astringent persimmon paste (AP) appearance. HM, high-methoxyl; LM, low-methoxyl.

Figure 3 shows images of pastes containing various polysaccharides, with the pure AP and gelatin-containing samples exhibiting rough, non-uniform textures, i.e., syneresis, whereas the samples containing HM pectin, LM pectin, guar, xanthan gum, carrageenan, or sodium alginate exhibit smooth textures. Upon heating (at 100 °C for 40 min or 121 °C for 4 min), the PAPP samples often developed rough textures, although the samples containing guar or xanthan gum retained their smoothness.

All pastes darkened after heating at 100 °C for 40 min and turned reddish after heating at 121 °C for 4 min.

These heat-induced color changes are ascribed to the oxidation of proanthocyanidins (Ikegami et al., 2009), which accumulate in the “tannin cells” of persimmon fruits during growth (Ikegami et al., 2007). Thus, the dark colors of the heated samples may be due to the solubilization of tannins and oxidation of proanthocyanidins during heating. After freezing, the pristine and gelatin-containing APs exhibit syneresis and non-uniformity, whereas the pastes containing HM pectin, LM pectin, guar, xanthan gum, carrageenan, or sodium alginate were smooth. Therefore, polysaccharides such as guar and xanthan gum maintain the smoothness of the paste even after heating or freezing.

**Figure 4:**
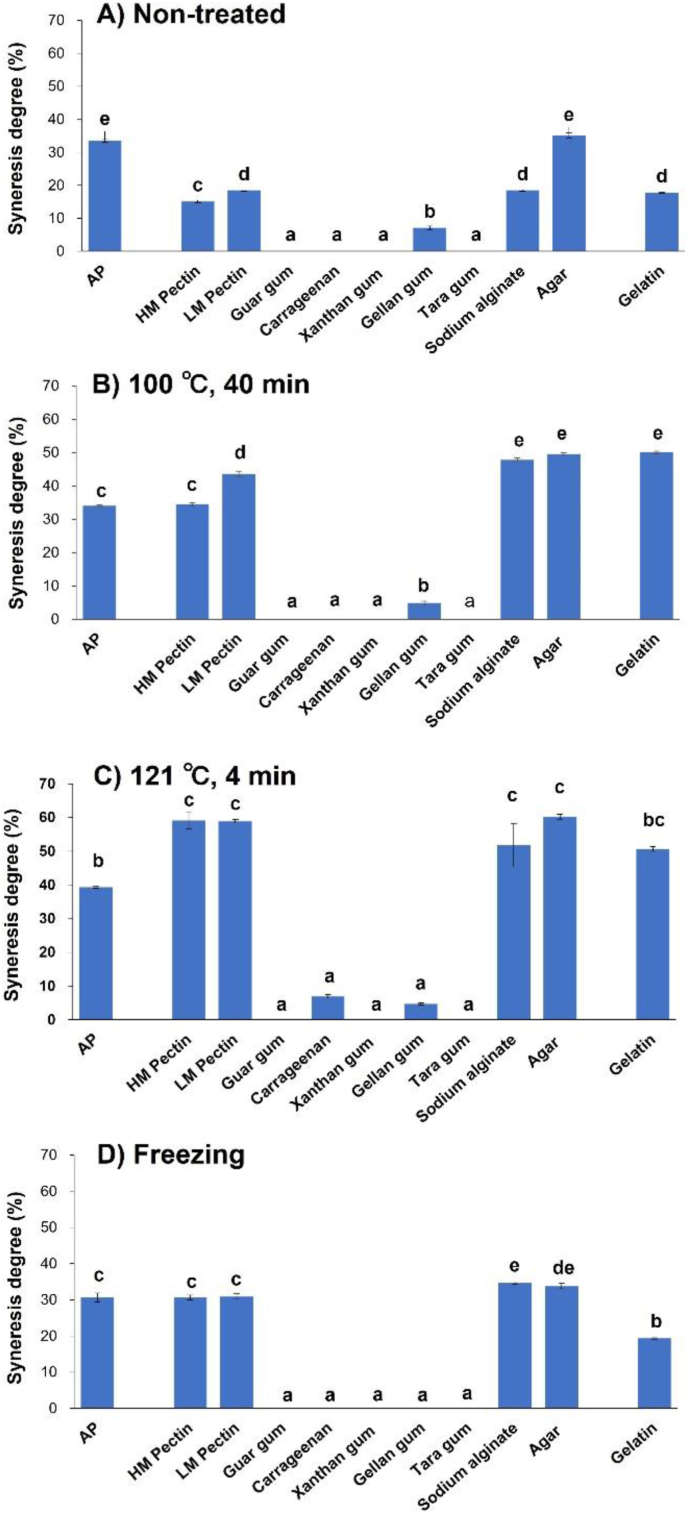
Effects of polysaccharide addition on the degree of syneresis of astringent persimmon paste (AP) subjected to (A) no treatment, heating at (B) 100 °C or (C) 121 °C, or (D) freezing. Vertical bars indicate mean values with standard errors (*n* = 3). Letters indicate significant differences (*P* < 0.05). HM, high-methoxyl; LM, low-methoxyl.

Given that the degree of syneresis of the pastes is difficult to determine directly, this parameter is calculated using the masses of the sample and supernatant obtained after centrifugation. The pristine and gelatin-containing APs exhibit significant syneresis (33.6 ± 0.5 and 17.7 ± 0.2, respectively) and are therefore poorly suited for use in processed products (Figure 4). Among the non-treated samples, those containing guar, xanthan, tara gum, carrageenan exhibited no syneresis, whereas low syneresis was observed for samples treated with gellan gum (7.1 ± 0.5). Meanwhile, high syneresis was observed for samples treated with HM (15.1 ± 0.4) and LM (18.4 ± 0.2) pectin, sodium alginate (18.5 ± 0.2), or agar (35.1 ± 0.7). For the pristine and gelatin-containing APs, significant syneresis was observed after all heat treatments. In contrast, samples containing guar, xanthan, tara gum or carrageenan exhibited no syneresis after heating at 100 °C for 40 min. Furthermore, no syneresis was observed for the samples with guar, xanthan, or tara gum even after heating at 121 °C for 4 min. After freezing, the results obtained for the samples with guar, xanthan, tara gum, or carrageenan were similar to those obtained for the corresponding non-treated samples, i.e., syneresis was inhibited. The high hydrophilicity and water retention properties of numerous polysaccharides are attributed to the presence of abundant hydroxyl and other hydrophilic groups and, in this study, improved the water retention characteristics and textures of the pastes studied.

## 4 Conclusions

Conventionally, the production of non-astringent persimmon paste involves a time-consuming, laborious astringency removal process, which generally includes treatment with dry ice or alcohol. Herein, we developed a simple, convenient method for removing the astringency of persimmon paste via polysaccharide addition. HM and LM pectins, carrageenan, xanthan gum, and sodium alginate exhibited high astringency removal efficiencies, and no astringency recurrence after freezing was observed for the samples containing HM pectin, LM pectin, guar gum, carrageenan, or sodium alginate. Moreover, HM pectin and sodium alginate effectively prevented astringency recurrence after heating. Guar, xanthan, tara gum, and carrageenan inhibited syneresis significantly well. Therefore, the original hypothesis was confirmed, i.e., high-quality pastes maintaining low astringency and syneresis after heating and freezing could be easily and efficiently produced by combining the individual favorable effects of multiple polysaccharides. However, the mechanism of polysaccharide-tannin binding remains unclear, although FT-IR and NIR analyses were conducted in this experiment, and thus, should be further investigated in future studies.

## Declarations

### Author contribution statement

Yoko Tsurunaga: Conceived and designed the experiments; Performed the experiments; Analyzed and interpreted the data; Contributed reagents, materials, analysis tools or data; Wrote the paper.

Tetsuya Takahashi, Mina Kanou: Performed the experiments.

Misaki Onda; Mika Ishigaki: Performed the experiments; Analyzed and interpreted the data; Wrote the paper.

### Funding statement

Professor Yoko Tsurunaga was supported by 10.13039/501100001691Japan Society for the Promotion of Science [21H00808 & 16K00814].

### Data availability statement

Data will be made available on request.

### Declaration of interest’s statement

The authors declare no conflict of interest.

### Additional information

No additional information is available for this paper.
